# Cuentos: A Large-Scale Eye-Tracking Reading Corpus on Spanish Narrative Texts

**DOI:** 10.1038/s41597-026-06798-z

**Published:** 2026-02-12

**Authors:** Fermin Travi, Bruno Bianchi, Diego Fernandez Slezak, Juan E Kamienkowski

**Affiliations:** 1 Laboratorio de Inteligencia Artificial Aplicada, Instituto de Ciencias de la Computación (Universidad de Buenos Aires – Consejo Nacional de Investigaciones Científicas y Técnicas), (C1428EGA), Buenos Aires, Argentina; 2 Departamento de Computación (Facultad de Ciencias Exactas y Naturales, Universidad de Buenos Aires), (C1428EGA), Buenos Aires, Argentina; 3 Maestría de Explotación de Datos y Descubrimiento del Conocimiento (Universidad de Buenos Aires), (C1428EGA), Buenos Aires, Argentina

**Keywords:** Language, Computational neuroscience

## Abstract

Eye-tracking is a well-established method for studying reading processes. Our gaze jumps word to word, sampling information almost sequentially. Time spent on each word, along with skipping or revisiting patterns, provides proxies for cognitive processes during comprehension. However, few studies have focused on Spanish, where empirical data remain scarce, and little is known about how findings from other languages translate to Spanish reading behavior. We present the largest publicly available Spanish eye-tracking dataset to date, comprising readings of self-contained stories from 113 native speakers (mean age 23.8; 61 females, 52 males). The dataset comprises both long stories (3300 ± 747 words, 11 readings per item on average) and short stories (795 ± 135 words, 50 readings per item on average), providing extensive coverage of natural reading scenarios with over 940,000 fixations covering close to 40,000 words (8,500 unique words). This comprehensive resource offers opportunities to investigate Spanish eye movement patterns, explore language-specific cognitive processes, examine Spanish linguistic phenomena, and develop computational algorithms for reading research and natural language processing applications.

## Background & Summary

The eyes have been proven to be a window into a wide range of cognitive processes, primarily related to attention and memory, both of which are fundamental for reading and reading comprehension. As such, the study of eye movements in reading dates back more than a century^1^ and has provided significant insights into language processing in the brain^2^. Reading patterns are actually non-sequential, as the eyes do not always move forward in a text, and sometimes we skip words altogether^3,4^. This behavior is primarily characterized by *saccades* and *fixations*^5^. Eye-tracking measures are derived from these fixations (or the lack of), as they get longer as the difficulty of the text increases, providing a measure of cognitive load.

Innovation in eye-tracking technology, combined with a reduction in costs, has allowed for extensive reading studies to be carried out. However, the majority of these studies have been conducted in English^6^, with a limited number of datasets available in other languages, including Chinese^7^, German^8^, Hindi^9^, Dutch^10^, and Russian^11^. Despite recent efforts to address the underrepresentation of many languages in eye-tracking research^6,12^, comprehensive and easy-to-access eye-tracking datasets during reading in Spanish remain notably limited. Furthermore, most Spanish studies have focused on single sentence reading^13–16^, and very few have studied natural reading of continuous text. One of the largest and most popular datasets in passage-level reading is the MECO dataset^6,17^, which offers data in Spanish from 48 Argentinian and 62 Chilean participants reading 12 Wikipedia-style texts (approximately 153,000 fixations and 690 unique words) and focuses on comparative studies between languages and translation. In contrast, we are releasing data from 113 participants reading 3 to 20 stories (30 unique stories total), with over 940,000 fixations and 8,500 unique words in total. This dataset is particularly suitable for artificial intelligence model training and in-depth studies due to its scale, the novelty of the Spanish texts included, and its standardized preprocessing and data structure. The consistency in processing criteria facilitates feature extraction for machine learning applications, while the standardized structure enables seamless integration of new participants and stimuli. The accompanying code suite further supports data validation, and automatic extraction and analysis of eye-tracking measures.

Applications for eye-tracking during reading span multiple domains, including cognitive science research on language processing^18^, educational assessment of reading comprehension and learning strategies^19^, clinical diagnosis of reading disabilities and neurological conditions^20–23^, and user experience design for digital interfaces^24^. More recently, however, these datasets have also begun to be explored in the field of natural language processing (NLP), where downstream tasks have been enhanced with information extracted from eye-tracking reading experiments with the goal of building more cognitively plausible computational models^25–27^. To this end, we combined eye-tracking data collected in previous studies on long^28,29^ and short^30^ stories in Spanish into a single, large, and comprehensive dataset, making it publicly available for the first time. To maximize the amount of data extracted, both studies based the selection of their stories on being self-contained, minimizing dialogues, as well as very short and very long sentences, and infrequent characters (such as punctuation marks, parentheses, or quotes), as these have a strong effect on eye-tracking measures and are usually discarded. Comprehension questions followed each reading in all trials to ensure attention, and a word association task with random nouns was also included in an attempt to remove any lingering bias between stories.

The extensive dataset collected presents diverse opportunities across multiple fields of study. In linguistics, it enables the investigation of Spanish-specific phenomena such as morphological complexity effects, word order preferences, and cross-linguistic comparisons with existing datasets in other languages. For psychological research, the data support studies on individual differences in reading strategies and the effects of text genre on cognitive processing. In NLP, data can enhance various tasks, including readability assessment, text simplification, and the development of more cognitively inspired language models.

## Methods

### Participants

The dataset comprises eye-tracking information from 113 participants (mean age 23.9 (IQR 4.8); 61 females, 52 males; mostly college students), collected in two previous studies^28–30^. All participants were native Spanish speakers, had normal or corrected-to-normal vision and each participated in only one of the two studies. They were recruited from the university mailing lists and were compensated with the equivalent of 5 USD per one-hour session. Written informed consent in agreement with the Declaration of Helsinki was provided by each of them. The data from two additional participants were discarded because Spanish was not their first language. The experiment was approved by the Comité de Ética del Centro de Educación Médica e Investigaciones Clínicas “Norberto Quirno” (CEMIC) (Protocol 435). Records were anonymized in compliance with ethical board approvals and contain no personal information.

### Corpus

Twenty and ten self-contained short (avg. 795 (±135), min. 680, max. 1220 words) and long (avg. 3300 (±147), min. 1975, max. 4640 words) stories written in Latin American Spanish were selected. From the twenty short stories, five were extracted from online Argentinian blog posts, and the other fifteen were extracted from “100 covers de cuentos clásicos”^31^ (see Table 1 for information on the stories and authors). These constitute classic stories that were simplified, translated (if necessary) and rewritten in Spanish by Hernán Casciari. The goal was to achieve diversity in literary style while maintaining consistency in both difficulty and slang. The long stories were extracted from several online sites^28,29^. In the case of non-public texts, explicit consent was obtained from each author to publicly release their stories.

**Table 1:** Text material.

Story	Corpus	Author	Genre	Readings	Words	Fixations
**Carta a una señorita en París**	A	J. Cortázar	Fantastic	6	2,582	17,271
**Sombras sobre vidrio esmerilado 1**	A	J. J. Saer	Realism	9	3,019	29,528
**Sombras sobre vidrio esmerilado 2**	A	J. J. Saer	Realism	8	2,331	19,922
**El loco cansino**	A	R. Fontanarrosa	Humor	10	2,568	26,229
**El negro de París**	A	O. Sariano	Adventure	11	3,509	38,629
**El origen de las especies**	A	C. Darwin	Science	11	3,783	47,546
**Axolotl**	A	J. Cortázar	Fantastic	11	1,543	21,014
**Carta abierta**	A	R. Walsh	Non-fiction	10	2,365	26,819
**Bienvenido Bob**	A	J. C. Onetti	Realism	16	2,423	44,120
**Rebeca**	A	O. Sacks	Non-fiction	18	2,377	46,722
**Cómo funciona caminar en la nieve**	B	V. Muro	Essay	45	1,066	47,302
**Las fotografías**	B	S. Ocampo	Fantastic	46	618	26,686
**Cómo funcionan los bolsillos**	B	V. Muro	Essay	46	972	45,815
**La máscara de la Muerte Roja**	B	E. A. Poe	Horror	47	572	26,641
**Educar para escalar y bucear**	B	A. Rieznik	Science	47	599	27,797
**Buenos Aires**	B	H. Casciari	Chronicle	47	607	28,813
**La noche de los feos**	B	M. Benedetti	Realism	49	544	25,774
**Embarrar la magia**	B	F. Alvarez Heduan	Essay	49	683	34,749
**El golpe de gracia**	B	A. Bierce	Realism	50	602	27,629
**La de la Obsesión por la Patineta**	B	J. Gallo	Contemp. Narrative	51	579	29,200
**La gallina degollada**	B	H. Quiroga	Horror	51	659	30,188
**Wakefield**	B	N. Hawthorne	Narrative	52	693	31,610
**La canción que cantábamos todos los días**	B	L. Lamberti	Horror	53	620	28,299
**Ahora debería reírme, si no estuviera muerto**	B	A. Carter	Magical Realism	53	606	25,629
**Rubí y el lago danzante**	B	M. Cohen	Sci-Fi	53	641	30,216
**La lluvia de fuego**	B	L. Lugones	Fantastic	53	640	30,960
**Una rosa para Emilia**	B	W. Faulkner	Gothic	55	643	33,946
**La salud de los enfermos**	B	J. Cortázar	Fantastic	56	667	34,486
**El almohadón de plumas**	B	H. Quiroga	Horror	56	579	28,063
**El espejo**	B	H. Murakami	Terror	56	628	29,851
**Total**				**1,125**	**39,757**	**941,454**

The description includes titles and original authors of each short story employed in the eye-tracking experiment; the total number of words included for computing gaze measures; the total number of readings per story (the overall number of participants was 113 but not all of them read all stories); and the total number of fixations (after data cleaning and used for computing gaze measures). Texts belonging to the long or short stories corpus (A and B respectively) are indicated in a separate column.

Selection criterion for the stories was based on not containing written dates and minimizing 1. dialogues, 2. very short and very long sentences (less than six words and greater than 29 words, respectively), 3. infrequent words (less than 100 appearances in the Latin American subtitles database EsPal^32^, and 4. infrequent characters (¿; ?; ¡; !; “;”; —; «; (;). The reasoning behind these criteria was to minimize the number of confounds in eye-tracking measures thereby maximizing the quality of the extracted information.

Additionally, the long stories feature cloze-predictability measures, which were estimated via a distinct online experiment^28^. For this experiment, participants were recruited from university mailing lists and social networks. Upon accessing a website and completing a personal information form (age, gender, native language, and reading skill), they were instructed to perform the classical cloze completion task. This task required reading stories in approximately 30-word sections and predicting the subsequent word. After entering their guessed word in a blank space and pressing enter, the subsequent ~30 words were displayed (along with the original word the participant had attempted to guess), featuring a new blank space for another word completion.

### Environment & setup

Both experiments used the same stimulation code programmed in MATLAB 2015a, with Psychtoolbox-3^33^; the code is available in the ‘Code Availability’ section. They took place in a dark room and employed the EyeLink 1000 (SR Research, Ontario, Canada) binocular eye-tracking device operating at 1000 Hz. The recordings of the short stories were done in a display with resolution 1920 × 1080, where participants sat at a distance of 55 cm, and the stimuli were presented in Courier New font, size 24, in black text on a gray background. The layout included 55-pixel line spacing, a left margin of 280 pixels, and a top margin of 185 pixels, allowing a maximum of fourteen lines per screen. Recordings belonging to the long stories, collected at an earlier date, followed the same setup, but at a lower resolution: 1024 × 768, sat at a distance of 65 cm, used font size 18, and the line spacing was 50 pixels. The margins were 150 and 120 pixels, respectively (ten lines per screen at maximum). Texts were divided across screens (typically between four and six for the short stories and between 24 and 59 for the long stories). Participants could navigate back and forth between them using the keyboard’s arrows. These events were logged by sending a message with a timestamp to the eye-tracker.

At the beginning of the experiment, participants were instructed to read the texts thoroughly, as they would be required to answer comprehension questions afterwards. They identified themselves using their initials and, in the case of the trials with short stories, rated their daily reading level on a scale from one to ten, with ten indicating over an hour of reading per day. In a subsequent stage of the study, participants were also asked about the time they went to sleep the previous night and their wake-up time. To make the instructions and keyboard controls clear, the first text displayed was a dummy text (dubbed ‘Test’), during which no eye-tracking was performed.

**Fig. 1:**
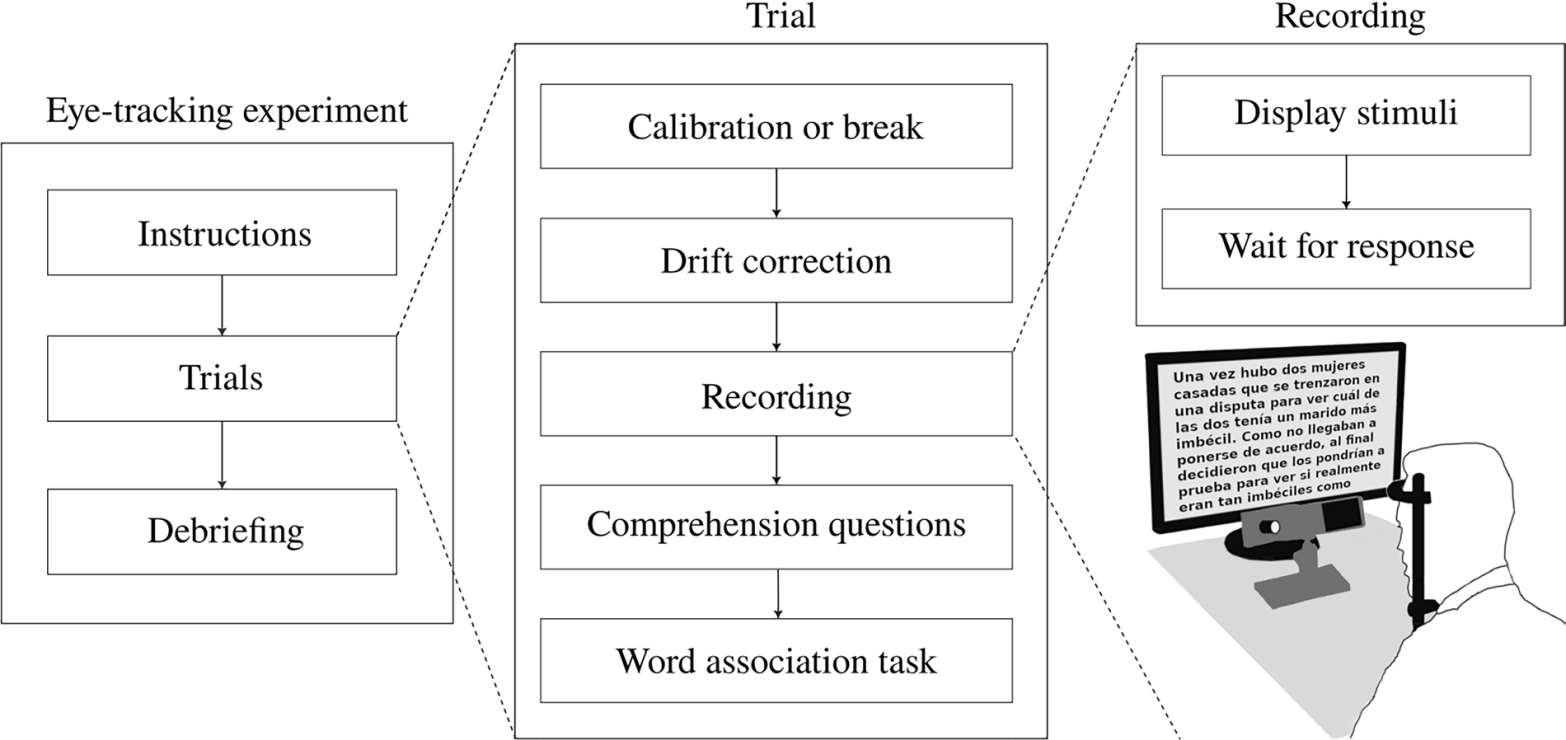
Depiction of the experiment setup, where each trial consisted of reading a short or long story. The story was divided into screens, and the participant was free to navigate back and forth between them. After completing each item, participants were asked to respond to comprehension questions about it. In the short stories trials, they were also required to complete a word association task, and 23 participants were asked two questions about their sleep and wake times from the previous night at the beginning of the experimental session.

Each story functioned as an experimental item and represented a separate trial. Before each trial, participants were asked whether they wanted to take a break, and eye-tracking calibration was performed before presenting the stimulus (Fig. 1). After calibration, participants had to fixate on a grid of nine points located at the screen’s margins and center both before and after the stimulus presentation, serving as a validation of the eye-tracker calibration. Items were shuffled randomly for each participant to achieve a similar number of readings of each text. Each participant read three to four long stories in a single two-hour-long session, with mandatory ten minute breaks in between. In the case of short stories, they were divided into two one-hour-long sessions of ten items each, with breaks of up to five minutes between items. Importantly, if a participant needed to leave before completing all ten trials, the following session would begin from the last read item. After completing the comprehension questions, participants who read the short stories engaged in a word association task, where individual words were displayed, and they had to write the first word that came to mind. For this task, five words were randomly selected from the 150 most frequent non-prepositions, non-verbs, and non-articles in the LexEsp corpus^32^, ensuring they did not appear in the stories. These same five words were consistently used for a given item across all participants. This task aimed to eliminate any residual bias from the reading activity before proceeding to the next item. Only after indicating they were ready could participants advance to the next trial.

### Data processing

All data were processed anew employing the custom code provided in this work. Given that the eye-tracker was used in binocular mode, fixations and saccades from both eyes were estimated using the Eyelink built-in algorithm. While both eyes are available in the raw data, processed data only include recordings from the eye that had the least calibration error as reported by the eye-tracker at the beginning of each trial. What constitutes a fixation, blink or saccade is predefined by the eye-tracker recording software with default parameters. We assigned a unique identifier, numbered sequentially, to each fixation throughout the entire trial. Since each item was divided into several screens, the timestamps of messages logged by the eye-tracker, when participants used the keyboard’s arrow keys to navigate between screens (i.e., sections of the story), were used to segment the recordings accordingly. From this division, we numbered the screen fixations sequentially. Initial and last fixations in each screen were automatically discarded, as well as very short and long fixations (under 50 ms or above 1000 ms), for they tended to correspond to wrong fixation detections or the participant not paying attention, respectively.

**Fig. 2:**
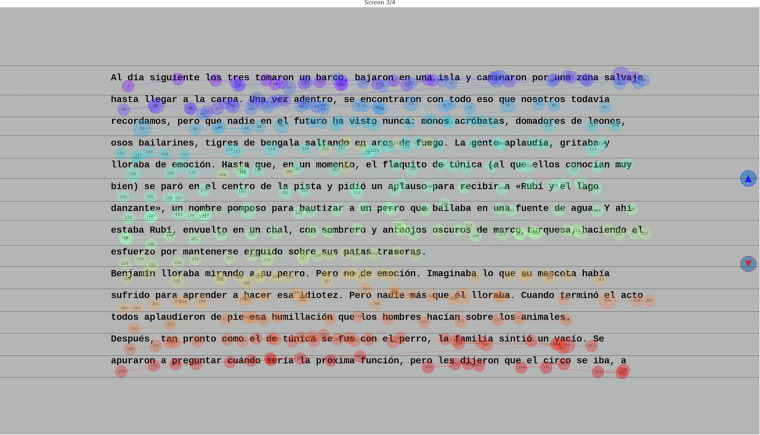
Depiction of one of the screens from a particular trial in the custom software employed. Each circle depicts a fixation, sequentially numbered and colored. Horizontal lines were manually positioned with the mouse to enclose rows of fixations and, in doing so, indicating what line of text each row of fixations corresponded to. These lines could all be moved up or down simultaneously using the buttons to the right. Artifactual fixations could also be removed with the mouse.

To derive eye-tracking measures, it is necessary to determine what fixations correspond to a given word. As the text is divided into lines, a crucial step is to bind a row of fixations to a given line. To achieve this, fixations were manually aligned to one of the closest lines, taking into account their proximity to the lines and the flow of the scanpathfixations were manually aligned to one of the closest lines, taking into account their proximity to the lines and the flow of the scanpath (Fig. 2). It was often the case that there was some miscalibration on the vertical axis, and fixations were manually adjusted vertically. In a few cases, fixations were shifted on the horizontal axis by a constant amount, and these were also corrected. However, if the calibration was very poor, trial data were excluded from further analysis. In some cases, participants returned to a previous screen by accident (indicated by a set of sparse fixations on random places in the screen), and these were removed. After manual processing, 121 of the 1250 trials were discarded due to poor calibration. The code used for this manual processing, as well as for computing the eye-tracking measures, is also available in the ‘Code Availability’ section and can be used for visually inspecting the data.

### Fixation assignment to words

Once the data were curated and horizontal lines were drawn below and above each text line in each screen, fixation assignment to words followed. Text lines were first split into words by using blank spaces as separators. A set of screen fixations was considered to belong to a given text line if their vertical axis fell within the (included) lower and (excluded) upper bounds of the corresponding horizontal lines.

When reading, as we move from one line to the next, our eyes usually do not fall precisely on the first word of the next line, and we make some additional fixations to adjust our eyes’ position. These are known as *return sweeps* and are considered the result of oculomotor errors, so they are discarded^34^. We computed them as any regressive fixation between the first and left-most fixation on a given text line. Additional care also had to be taken with fixations resulting from returning to a previously seen screen. These fixations were numbered starting from the last fixation number on that screen.

After these steps were carried out, the fixations corresponding to a given word are those whose horizontal coordinates fall within the word’s surrounding blank spaces. The right blank space is excluded, which means it is included in the following word, as we tend to read from left to right.

### Eye-tracking measures

The following exclusion criteria were applied when calculating eye-tracking measures for a given word in a trial: no measures were computed if the word was first or last in a sentence, first or last on a line, or contained any of the following characters: (¿;, ‘?’; ‘¡’, ‘!’, ‘.’, ‘−’, ‘1’, ‘2’, ‘3’, ‘4’, ‘5’, ‘6’, ‘7’, ‘8’, ‘9’, ‘0’. It is important to note that these criteria are applied at the item level, meaning the excluded words are consistent across all trials.

To calculate these measures, we define a *regression* as consisting of any re-fixation on a word after exiting it, either to the right or left. ‘Exiting’ a word includes skipping it. Under this definition, first-fixation duration, single-fixation duration, and gaze duration are only defined if a fixation enters the word from an earlier region of text before a fixation occurs on a later region of text in that screen^35^. Fixations originating from returning screens are considered regressions if either the word they land on or any of the following words in the screen were already fixed.

These measures are extracted on a trial-by-trial basis and are typically categorized as ‘early,’ ‘intermediate,’ or ‘late,’ depending on the stage of reading processing they represent (Table 2). Early measures primarily reflect automatic processes such as word recognition and lexical access, whereas late measures are more indicative of deliberate, controlled, and strategic processing^36,37^. ‘Likelihood of skipping’ (or *skipping rate*) and ‘regression rate’ are exceptions in that they are calculated across trials rather than within individual trials.

**Table 2:** Eye-tracking measures extracted from trials, categorized as early, intermediate, or late depending on which processing stage of reading they reflect.

Early measures	
**First Fixation Duration (FFD)**	Duration of the first, and only the first, fixation on a word before exiting (either to the right or to the left).
**Single Fixation Duration (SFD)**	Duration of the first and only fixation on a word (equal to FFD for single fixations). Zero if the word has more than one fixation.
**First Pass Reading Time / Gaze Duration (FPRT / GD)**	Sum of all fixations on a word before exiting (either to the right or to the left).
**Likelihood of Skipping (LS)**	Number of first-pass fixations divided by the number of trials.
**Intermediate measures**	
**Regression Path Duration (RPD)**	Time spent re-reading previous words before exiting the area of interest to the right.
**Regression Rate (RR)**	Number of trials with a regression divided by the number of trials.
**Late measures**	
**Total Fixation Duration (TFD)**	Sum of all fixations on a word, including regressions.
**Re-Reading Time (RRT)**	Regression path duration minus first pass reading time.
**Second Pass Reading Time (SPRT)**	Sum of fixations after a word has been exited for the first time.
**Fixation Count (FC)**	Number of fixations on a word.
**Regression Count (RC)**	Number of regressions made on a word.

## Data Records

The dataset, both processed and raw, can be found in Figshare^38^. It is divided into folders (one for each participant), and inside each folder is the data from the corresponding participant trials. The stimuli displayed can be found in the ‘stimuli’ folder, and their order, comprehension questions, and configuration are under the ‘metadata’ folder. We recommend using the code provided to explore this data (see ‘Usage notes’).

Raw data files pertain to the eye-tracker recordings (in EDF and ASCII formats) and to the participant and trials’ metadata (structures saved in.mat). Participant metadata includes the following fields:

- *subjname*: Participant anonymized name (e.g. sub-001).
- *reading_level*: Daily reading score (scale from 1 to 10, or ‘NA’).
- *use_eyetracker*: Indicates if the eye-tracker was used.
- *shuffled_stimuli*: List of the stimuli order.
- *stimuli_index*: Last story read in the list.
- *n_sessions*: Number of sessions.
- *fst_sleeptime*: Time of sleep in the first session (or ‘NA’).
- *snd_sleeptime*: Time of sleep in the second session (or ‘NA’).
- *fst_wakeuptime*: Wake-up time in the first session (or ‘NA’).
- *snd_wakeuptime*: Wake-up time in the second session (or ‘NA’).

Trial metadata includes the following:

- *subjname*: Participant anonymized name (e.g. sub-001).
- *stimuli_index*: Order of the stimulus in the experiment.
- *file*: Path to the file displayed.
- *sequence*: Sequence of screen indices with timestamps.
- *questions_answers*: Answers to the comprehension questions.
- *synonyms_answers*: Answers to the word association task.

Processed data, on the other hand, are available as binarized Dataframes from Python’s Pandas library in Pickle format (.pkl). Inside each participant’s folder, there is a copy of their metadata (named ‘profile.pkl’) and a folder for each of their trials. These folders contain all trial metadata in separate files and a file (‘flags.pkl’) with processing information (whether it was edited, if the first or last validation points were not fixated properly, number of wrong answers in the comprehension questions, if the trial was wrong and excluded from further analysis, and to what session it belongs to). Additionally, trial fixations (‘fixations.pkl’) are divided into folders according to which screen they belong to, with a separate file (‘lines.pkl’) that indicates the horizontal lines that determine which text line each fixation belongs to. Calibration files containing the grid (‘cal_points.pkl’) and resulting validation from the calibration procedure (‘val_offset.pkl’, ‘val_points.pkl’), alongside the fixations on the manual validation points (‘first.pkl’, ‘last.pkl’, corresponding to the beginning and ending of the trial, respectively) are also provided and saved in separate folders. These can be observed using the custom code provided and are useful for determining whether the eye-tracker’s calibration was off at the start or end of the trial.

Cloze-task data for the long stories corpus are provided in the form of separate CSV files inside the folder ‘cloze_task’, under the data directory. There is one row per word with its corresponding human predictability estimate.

Of the 113 participants, 76 of them took part in the short-story experiment, resulting in an average of 50 participants per story, while the long stories had an average of 11 participants per story. Sleep and wake times from the previous night were provided by 23 participants, covering a total of 330 trials.

## Technical Validation

Analysis of linguistic data is most commonly conducted through linear mixed-effects (LME) models^39^. The advantages of LME models stem from the fact that every data point is included in the analysis (there is no averaging, as in ANOVA) and the lack of need to run separate analyses for items and participants, as they are both included as random variables in the same analysis. Control variables (covariates), such as word length or frequency, can be included in these models.

In the case of word measures derived from eye-tracking during reading (e.g. first-pass reading time, or FPRT), they are usually set as the dependent variable in LME models. Word location in the item, sentence and screen are all included as independent variables (fixed effects, known to influence these measures). Word length and frequency are added as covariates, and participants and items (stimuli) are added as random effects (to account for individual variation in both participants and the items chosen). The resulting formula for FPRT is presented in Eq. 1.

1 $$\begin{array}{c}{FPRT} \sim {WordLength}\ast {WordFrequency}+{ItemPosition}+{SentencePosition}\\ \,+\,{SentencePosition}2+{ScreenPosition}+(1{|Participant})+(1{|Item})\end{array}$$

To perform this analysis, eye-tracking measures are first log-transformed to reduce skewing in the data and covariates are centered with respect to their mean. Word frequency is obtained from the EsPal corpus^32^ and is also log-normalized; words with no frequency information are discarded from the analysis. Consistent with the bibliography, word length is expressed as one over the number of letters. Finally, as skipped words have a first fixation of 0 ms by definition, they are excluded from this analysis. First fixation duration (FFD) is analysed with the same processing steps, excluding word position in the sentence or on the screen, as it did not yield statistical significance. Skipping, being a categorical variable (i.e., a word is either ‘skipped’ or ‘not skipped’), is handled with a binomial LME model, with word position both in the item and screen added as fixed effects.

Both FFD and FPRT are significantly affected by word length: the negative estimate indicates that, as word length decreases, both of these measures decrease (Tables 3, 4). Word frequency also significantly affects both measures, increasing FFD and FPRT as the word’s frequency decreases, though its effect on FPRT is notably small (estimate = 0.001). Additionally, the interaction between word length and frequency (WordLength:WordFrequency row) is significant for both measures. Its positive estimate indicates that the effect of word length on these measures increases as word frequency increases, with a more prominent interaction in FPRT (meaning that shorter words tend to be processed even faster when they are common)^8,18,28,29,40^. Word position in the item also interacts significantly with both of these measures, while its position in the sentence and screen has a significant effect on FPRT only (these effects, however, are weaker than word length or frequency, as indicated by the absolute value of the t-stat). In the case of FPRT, the squared sentence position has a positive interaction with it, implying that words at the beginning and end of sentences increase this measure^28,29^.

**Table 3:** Results of the LME model on FFD as the dependent variable.

Fixed effect	Estimate	CI	t-stat	P-val
**(Intercept)**	5.340 (0.010)	[5.320; 5.358]	542.01	<0.001
**WordLength**	−0.055 (0.007)	[−0.068; −0.041]	−8.09	<0.001
**WordFrequency**	−0.005 (0.000)	[−0.006; −0.004]	−13.07	<0.001
**ItemPosition**	−0.023 (0.002)	[−0.027; −0.020]	−13.71	<0.001
**WordLength:WordFrequency**	0.01 (0.002)	[0.013; 0.020]	9.72	<0.001
**Random effect**	**Var**	**Std**		
**Participant (Intercept)**	0.010	0.10		
**Item (Intercept)**	0.000	0.01		
**Residual**	0.132	0.36		

CI corresponds to the lower (2.5%) and upper (97.5%) bounds of the 95% confidence interval for the estimate.

**Table 4:** Results of the LME model on FPRT as the dependent variable.

Fixed effect	Estimate	CI	t-stat	P-val
**(Intercept)**	5.364 (0.011)	[5.343; 5.386]	488.89	<0.001
**WordLength**	−0.451 (0.008)	[−0.466; −0.436]	−58.87	<0.001
**WordFrequency**	0.001 (0.000)	[0.000; 0.002]	2.43	0.015
**SentencePosition**	−0.062 (0.008)	[−0.078; −0.046]	−7.80	<0.001
**SentencePosition²**	0.050 (0.009)	[0.032; 0.069]	5.35	<0.001
**ItemPosition**	−0.028 (0.002)	[−0.032; −0.024]	−14.28	<0.001
**ScreenPosition**	−0.008 (0.002)	[−0.012; −0.004]	−4.09	<0.001
**WordLength:WordFrequency**	0.113 (0.002)	[0.109; 0.117]	58.85	<0.001
**Random effect**	**Var**	**Std**		
**Participant (Intercept)**	0.012	0.11		
**Item (Intercept)**	0.000	0.02		
**Residual**	0.170	0.41		

CI corresponds to the lower (2.5%) and upper (97.5%) bounds of the 95% confidence interval for the estimate.

Concerning Skipped words (Table 5), word length and frequency increase its probability, as indicated by their positive estimate and significant effect. Word length, particularly, has a high estimate and, thus, a large impact on the measure, suggesting even a small decrease in word length drastically increases the log-odds of skipping the word. The high negative estimate in the interaction between word length and frequency suggests that for frequent words, length is less critical in the decision to skip, whereas for less frequent words, shorter length significantly increases the likelihood of skipping. These results also fall in line with established literature^4,40^. Finally, as in the case of FPRT, word position on the screen and item interact significantly with the likelihood of skipping a word.

**Table 5:** Results of the logistic ME model on Skipped as the dependent variable.

Fixed effect	Estimate	CI	Z-stat	P-val
**(Intercept)**	−0.108 (0.046)	[−0.198; −0.019]	−2.38	0.018
**WordLength**	4.959 (0.033)	[4.894; 5.024]	149.61	<0.001
**WordFrequency**	0.097 (0.009)	[0.094; 0.101]	52.84	<0.001
**ItemPosition**	0.112 (0.008)	[0.105; 0.139]	14.22	<0.001
**ScreenPosition**	0.101 (0.009)	[0.084; 0.118]	11.824	<0.001
**WordLength:WordFrequency**	−0.545 (0.009)	[−0.562; −0.527]	−61.10	<0.001
**Random effect**	**Var**	**Std**		
**Participant (Intercept)**	0.209	0.46		
**Item (Intercept)**	0.007	0.08		

CI corresponds to the lower (2.5%) and upper (97.5%) bounds of the 95% confidence interval for the estimate.

## Usage Notes

The code provided in the section below is divided into three parts: MATLAB code for the experiment, Python code for visualization and supervised data curation, and Python code for the computation and subsequent analysis of the eye-tracking measures. The entry point for running the experiment is run_experiment.m, although it is primarily left for reference, and there is no guarantee of its correct execution.

To run the Python code, download the dataset from Figshare^38^ and place it in the root folder of the git repository. To visualize and/or edit the data, run the script edit_trial.py. If you wish to inspect the raw data, remove the ‘*processed’* folder from the ‘*data’* directory. To compute and analyse the eye-tracking measures, run the script em_analysis.py. The necessary packages can be installed via *pip* using the requirements.txt file. Linear Mixed Models analysis is performed using the *pymer* package, which requires having R installed (see the package installation notes for further instructions).

## Data Availability

The dataset is available at 10.6084/m9.figshare.28311908.

## Code availability

The code necessary for inspecting the data and running the different analyses can be found at https://github.com/NeuroLIAA/reading-et. Python 3.10 or greater is required, with the necessary packages being listed in the requirements.txt file. The software used for converting the raw EDF files to ASCII was EDF2ASC version 4.2.762.0 Linux, part of the EyeLink Display Software.
